# Andrographolide Inhibition of Th17-Regulated Cytokines and JAK1/STAT3 Signaling in OVA-Stimulated Asthma in Mice

**DOI:** 10.1155/2021/6862073

**Published:** 2021-05-29

**Authors:** Qian Yu, YaJie Shi, Chang Shu, XuChun Ding, ShiPing Zhu, ZhouPing Shen, YaFang Lou

**Affiliations:** Respiratory Department, Hangzhou TCM Hospital Affiliated to Zhejiang Chinese Medical University, Hangzhou 310000, China

## Abstract

Asthma has long been considered a disease of airway inflammation. The excessive or prolonged production of inflammatory mediators can result in airway remodeling and severe clinical syndromes such as dyspnea or even apnea. Therefore, pharmaceutical intervention is required to restrain the excessive release of such inflammatory mediators in control of asthma. Novel therapeutics and mechanistic insight are sought for the management of this chronic inflammatory disease. Andrographolide (AG) is a type of diterpenoid ester compound and is reported to demonstrate multiple properties such as antioxidation and anti-inflammation. However, the anti-inflammatory capacity of AG by regulating immunologic function in airway of asthma has not been fully studied to date. Therefore, this study investigates whether AG is capable of suppressing the inflammatory response of asthma in OVA-stimulated mice and the mechanism by which this is achieved. Animals were randomly divided into 4 groups: control group, OVA model group, OVA + AG (0.1 mg/ml) group, and OVA + dimethylsulfoxide (DMSO) group. The serum, BALF, and lung tissue of the mice were collected separately for the administration of ELISA, rt-PCR, western blot and pathological section and staining. We found that AG attenuated the OVA-induced production of IL-6, IL-17A, IL-17F, and ROR*γ*t; inhibited the OVA-mediated phosphorylation of JAK 1 and STAT3; and alleviated airway remodeling and the neutrophil infiltration of lung tissue. We conclude that AG inhibits the inflammatory response of asthma in OVA-stimulated mice by blocking the activation of Th17-regulated cytokines and the JAK1/STAT3 signaling pathway.

## 1. Introduction

Asthma has long been considered the disease of chronic airway inflammation with bronchial hyperresponsiveness. It usually presents with repeated airway spasm which could be relieved spontaneously or by using bronchodilators. The global asthma report indicated that more than 300 million people suffered from asthma [1]. It is investigated that there are approximately 30 million asthmatic patients in China while the number is still escalating year by year [2]. Asthma hugely undermines people's health. Although nowadays there are drugs such as inhaled corticosteroids, bronchodilator, and leukotriene receptor antagonist, which could decrease the acute attack of asthma to a certain extent, these drugs could hardly control and cure all of the asthmatic patients along with causing side effects in the meantime.

Andrographolide (AG) is a kind of diterpenoid ester compound which is extracted from the herb or leaves of Andrographis paniculata as one of the effective components [3]. It proves that AG plays a vital role in the treatment of multiple diseases such as asthma, chronic obstructive pulmonary disease, idiopathic pulmonary fibrosis, hepatitis, cirrhosis, neurodegenerative diseases, and autoimmune diseases with the function of anti-inflammation and antioxidation [4]. The mechanism of AG in treating asthma has been explored by many researches. It revealed that AG reduced the total white blood cells especially the eosinophils in the bronchoalveolar lavage fluid (BALF) of ovalbumin- (OVA-) induced asthmatic mice to relieve the inflammatory reaction and mucous secretion and finally lowered the airway hyperresponsiveness by inhibiting nuclear factor kappa-light-chain-enhancer of activated B (NF-*κ*B) pathway [5, 6]. AG also hindered the activation of neucleotide-binding oligomerization domain, leucine-rich repeat and pyrin domain-containing (NLRP) 3 inflammasome and eliminated the production of reactive oxygen species [5]. Moreover, AG could inhibit the phosphorylation of histone deacetylase 2 and the expression of inflammatory mediator such as interleukin (IL)-27 by blocking phosphatidylinositol 3 kinase (PI3K)/protein kinase B (AKT) pathway in steroid-resistant airway hyperresponsive mice models stimulated by lipopolysaccharide and Interferon-c [7]. The researches above manifested that AG functioned through mediating inflammatory action, oxidative stress, and epigenetics. Asthma is not only a kind of chronic airway inflammatory disease, but also an allergic disease with abnormal immunity. It is still not clear if AG could play a role by regulating immunologic function.

T helper (Th) 17 cells are a new subset of immune cells which play important roles in many diseases like tumor and autoimmune diseases [8]. As a member of CD4 + T cells, Th17 cells express many cytokines such as IL-17A, IL-17E, IL-17F, and IL-22 [9, 10]. It has been concluded that the activation of Th17 cells was one of the pathogenesis of asthma: asthmatic patients expressed elevated mRNA and protein of IL-17A which could be closely related to airway hyperresponsiveness [11–13]. IL-17 F gene polymorphism could lower the risk of asthma by malfunctioning IL-17F [14]. Researches also showed that the stimulation of bronchial epithelial cells and fibroblast by IL-17A and IL-17F caused the recruitment and activation of neutrophils which could increase the secretion of multiple cytokines, extracellular matrix proteases and elastases to trigger inflammation, airway hyperresponsiveness, and mucus squamous differentiation [15, 16]. Th17 cells could expedite the proceedings of airway remodeling by the superabundant collagen synthesis and the accumulation of extracellular matrix led by stimulating myofibroblast (MF). All of these pathological products could enhance the expression of actins in airway smooth muscle (AMS) cells to promote the proliferation. In this study, we explored the resistant effects of AG in regulating airway remodeling and inflammatory cells infiltration.

As a kind of nonreceptor tyrosine protein kinase, phosphorylation of Janus kinase (JAK) 1 could be activated and phosphorylated by the stimulation of plentiful pathogens. Signal transducer and activator of transcription (STAT) 3 is positioned downstream of JAK1 and can be activated via phosphorylation. Once activated, the dipolymer of STAT3 is formed and translocated into the nucleus to regulate target genes [17]. ROR*α* and ROR*γ*t are considered to be the nuclear receptors and master regulators mediating the differentiation of Th17 cells which have been implicated in the pathology of several inflammatory diseases as inflammatory bowel disease, autoimmune arthritis, and skin pathologies [18–20]. In in vitro research, ROR*α* and ROR*γ*t coexpression has been proved to synergistically promote Th17 differentiation. Therefore, in this study we investigated the inhibitory effects and the underlying mechanism of AG on the differentiation of Th17 cells in OVA-induced asthmatic mice through JAK1/STAT3 signaling and its targeted nuclear receptors.

## 2. Materials and Methods

### 2.1. Animals and Care

40 male BALB/c mice, 6–8 weeks, weighing 20–25 g, were purchased from Laboratory Animal Centre of Zhejiang University of Traditional Chinese Medicine (SYXK[Zhe] 2013–0184). All animal procedures performed in this study were approved by the Institutional Animal Care and Use Committee of ZheJiang University of Traditional Chinese Medicine (No. 20170072). The mice were maintained in a specific pathogen-free (SPF) environment under controlled conditions of temperature (22 ± 1°C), humidity (50%), 12 h light-dark cycles, and free access to food and water. All efforts were made to ameliorate the welfare and minimize animals suffering.

### 2.2. Chemicals and Antibodies

Andrographolide (C_20_H_30_O_5_) was purchased from Dalian Meilun Biotechnology Co., Ltd. (Dalian, China). The sample consisted of a white powder with a molecular weight of 350.45 and a purity of above 98% determined by HPLC analysis. OVA was purchased from Dalian Meilun Biotechnology Co., Ltd. (Dalian, China). The aluminum hydroxide gel was purchased from Thermo Fisher Scientific (Watham, MA, USA). The IL-6, IL-17A, and IL-17F ELISA kits were purchased from Multi Sciences (Hangzhou, China). Primary antibodies and HRP-conjugated anti-rabbit IgG were purchased from Bioworld Technology (Minnesota, USA), Cell Signaling Technology (Beverly, MA, USA), Affinity Bioscience (Shanghai, China), and Proteintech Group (Wuhan, China), respectively. SuperSignal West Femto Stable Peroxide Substrate and AlexaFluor594-labeled goat anti-rabbit IgG antibodies were obtained from Thermo Scientific (San Jose, CA, USA).

### 2.3. Animal Groups and Model

Animals were randomly divided into 4 groups (10 in each group): control group, OVA model group, OVA + AG (0.1 mg/ml) group, and OVA + dimethylsulfoxide (DMSO) group (DMSO is the solvent of AG). The OVA model was established according to the method described in a previous study [21]. Animals were sensitized by intraperitoneal injection of 20 *μ*g OVA in 200 *μ*L and an equal volume of aluminum hydroxide on day (D) 1 and D 14, respectively. Starting 21 days after the second sensitization, mice were challenged with atomized OVA (1% OVA dissolved into phosphate-buffered saline-PBS) for 30 min each day for 7 days. The mice were intraperitoneally administrated with AG 1 h before each challenge while the mice in control group were intraperitoneally treated with 0.9% physiological saline.

### 2.4. Sample Collection

At 24 h after the last drug administration, the mice were anesthetized by intraperitoneal injection of pentobarbital sodium (40 mg/kg). BALF of the mice was collected immediately using PBS lavaged to the right lung 3 times by gentle cannulation. The BALF was centrifuged and the supernatant was stored at −80°C for ELISA. Blood samples were collected into centrifuge tubes, stored for 2 h at 4°C, and subsequently centrifuged at 3000 × g at 4°C for 10 min. The serum was stored at −80°C for ELISA. The lung tissues were collected. The left lung was fixed in 10% neutral formalin overnight for HE, Masson stainings. The right lung was stored in liquid nitrogen until extraction for RNA and protein.

### 2.5. Enzyme-Linked Immunosorbent Assay (ELISA)

The levels of IL-6, IL-17A, and IL-17F in the BALF supernatant and serum were determined using ELISA following the manufacturer's instructions.

### 2.6. Histologic Evaluation

The left lung was immersed in 10% neutral formalin overnight. Part of the lung tissues were then sectioned, dewaxed, dehydrated in decreasing concentrations of ethanol, rinsed with distilled water, and stained separately using hematoxylin and eosin for HE staining and Masson staining fluid for Masson staining. Some of the sections were blocked with serum and incubated with primary antibodies in 4°C overnight. On the next day, the sections were incubated with secondary antibodies in 37°C for 20 min and visualized with DAB (ZSGB-BIO, Beijing, China) and Masson staining fluid (Baso Diagnostic Inc., Zhuhai, China). Microscopic images of stained sections were obtained using the Nikon eclipse 80i microscope (Nikon, Japan) at ×250 and 500 magnification and scanned by NanoZoomer 2.0 RS (Hamamatsu, Japan).

### 2.7. RNA Isolation and Real-Time Polymerase Chain Reaction (PCR)

The total RNA was extracted using an RNA Extraction Kit (Takara Bio, Japan). Reverse transcription was performed with a TIANScript RT Kit (Takara Bio, Japan) to obtain cDNA. Real-time PCR was performed on an ABI StepOnePlus real-time PCR instrument (Bio-Rad, California, USA) with the SYBR Green qPCR SuperMix (Takara Bio, Japan). The transcripts were amplified in one tube containing 1.5 *μ*l of cDNA and 0.5 *μ*l of each of the forward and reverse primers. PCR amplification was performed at 95°C for 3 min followed by 40 cycles at 95°C for 10 s and 60°C for 30 s. The melting curve analysis was conducted after amplification to verify the accuracy of the amplicon. It was performed at 55°C. The temperature rose 0.5°C every 30 s till 95°C by one cycle. Primer sequences for qPCR of retinoic-acid- (RA-) related orphan receptor *γ* thymus (ROR*γ*t), RA-related orphan receptor *α* (ROR*α*), and *ß*-actin mRNA are shown in Table 1.

### 2.8. Western Blot Analysis

Total tissue protein was isolated with RIPA buffer (50 Mm Tris–HCl, 300 mM NaCl, 0.5% TritonX-100, 5 mM EDTA, cocktail protease inhibitor), separated by SDS-PAGE and transferred to polyvinylidene fluoride (PVDF) membranes. The membranes were blocked with 5% nonfat milk and incubated with primary antibodies in 4°C overnight. On the next day, the membranes were washed with PBST (phosphate-buffered saline with 0.1% Tween 20) for three times, incubated with secondary antibodies at room temperature for 1 h, washed with PBST for another three times, and visualized with Immobilon Western Chemiluminescent HRP Substrate. The relative optical densities were scanned with Odyssey Infrared Imaging System (LI-COR, Odyssey CLx, USA).

### 2.9. Statistical Analysis

The data are represented as the mean and the calculated standard deviation for each group. The results were assessed by a one-way analysis of variance with SPSS statistical software. *p* < 0.05 was considered to be statistically significant.

## 3. Results

AG inhibited the production of cytokines expressed by Th17 cells and the expression of transcription factor induced by Th17 cells in OVA-stimulated asthmatic mice.

**Figure 1:**
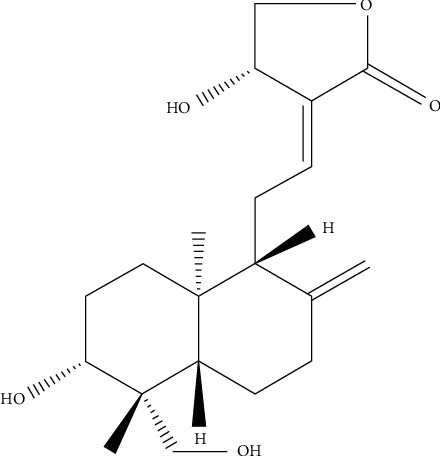
Chemical structure of Andrographolide (C20H30O5).

**Figure 2:**
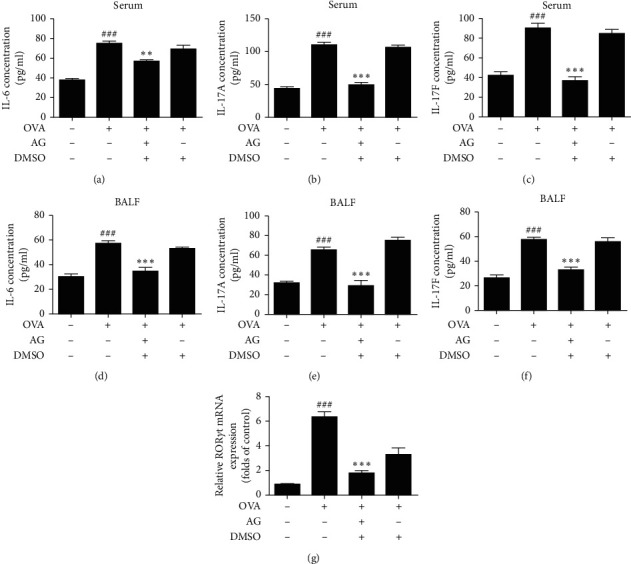
AG inhibited the production of cytokines expressed by Th17 cells and the expression of transcription factor induced by Th17 cells in OVA-stimulated asthmatic mice. Animals were randomly divided into 4 groups, sensitized by intraperitoneal injection of OVA (0.1 *μ*g/*μ*L) and intraperitoneally administrated AG (0.1 mg/ml) 1 h before each challenge with atomized OVA. The serum and BALF were collected and used to detect the concentration of IL-6, IL-17A, and IL-17F separately. The production of IL-6, IL-17A, and IL-17F was inhibited by AG (a–f). The lung tissues were collected and used to detect the mRNA expression of ROR*α* and ROR*γ*t. The mRNA expression of ROR*γ*t (g) was inhibited by AG. All values are expressed as the mean ± the standard deviation of triplicate tests. ^###^*p* < 0.01 relative to the control group; ^*∗∗∗*^*p* < 0.01 and  ^*∗∗*^*p* < 0.01, relative to the OVA group.

The chemical structure of AG is shown in Figure 1. We used the concentration of 1 mg/kg of AG during the following experiments. As shown in Figures 2(a)–2(f), the stimulation with OVA (0.1 *μ*g/*μ*L) increased the production of IL-6, IL-17A, and IL-17F in both serum and BALF of the mice while it was significantly inhibited by AG. Next, we also detected the effect of AG on the mRNA levels of ROR*α* and ROR*γ*t by RT-PCR. We found that OVA stimulation upregulated ROR*α* and ROR*γ*t gene expression. Expectedly, AG decreased ROR*γ*t mRNA expression in OVA-stimulated asthmatic mice (Figure 2(g)). But we did not get the same result of ROR*α* mRNA expression.

There was an AG inhibition of the JAK1/STAT3 signaling pathway and the downstream target protein in OVA-stimulated asthmatic mice.

**Figure 3:**
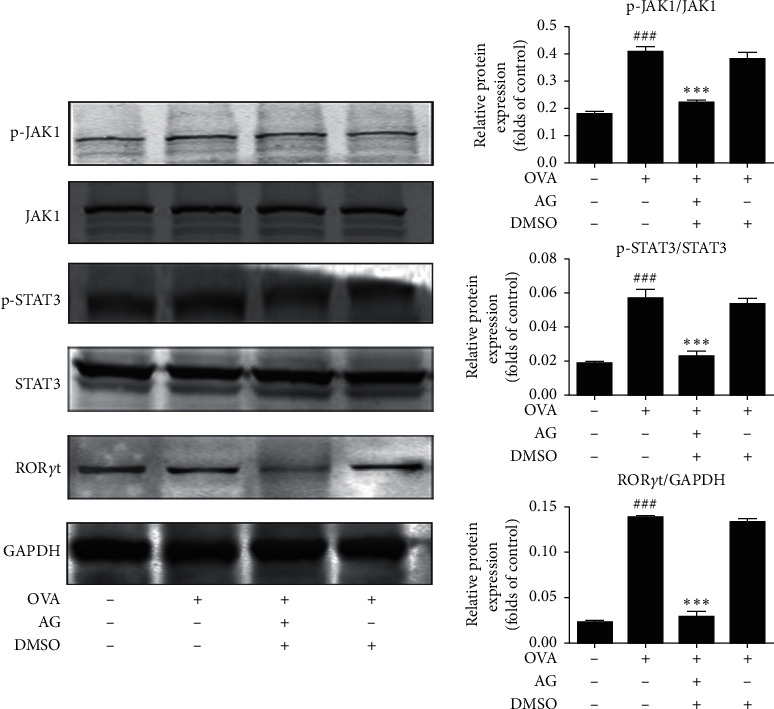
AG inhibited JAK1-STAT3 activation and the expression of transcription factor in OVA-stimulated asthmatic mice. As shown in Figure 3, OVA (0.1 *μ*g/*μ*L) treatment induced JAK1 and STAT3 phosphorylation, which were abated by AG in the dosage of 0.1 mg/ml (*p* < 0.01). Furthermore, the expression of the downstream transcription factor ROR*γ*t was also inhibited by AG in OVA-induced asthmatic mice (*p* < 0.01). The values are expressed as the mean ± the standard deviation of triplicate tests. ^###^*p* < 0.01 relative to the control group; ^*∗∗∗*^*p* < 0.01, relative to the OVA group.

We next investigated the effect of AG on the upstream JAK1/STAT3 signaling pathway. As shown in Figure 3, OVA (0.1 *μ*g/*μ*L) treatment markedly induced JAK1 and STAT3 phosphorylation, which was significantly ablated by AG (*p* < 0.01). We also verified the effect of AG on the expression of downstream transcription factors. As shown in Figure 3, OVA (0.1 *μ*g/*μ*L) intervention induced ROR*γ*t phosphorylation, which was also significantly inhibited by AG (*p* < 0.01). However, we did not get the same result on the expression of ROR*α*.

There was an AG inhibition of the neutrophil infiltration of lung tissue in OVA-stimulated asthmatic mice.

**Figure 4:**
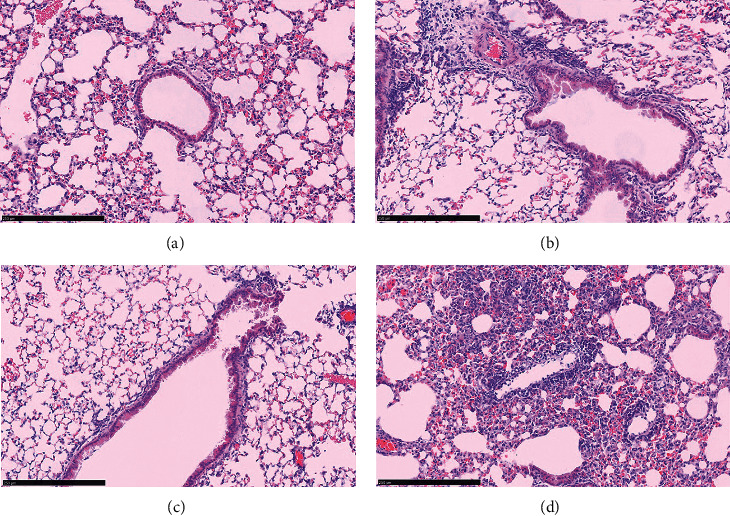
AG inhibition of the neutrophil infiltration of lung tissue in OVA-stimulated asthmatic mice. Animals were randomly divided into 4 groups: control group (a), OVA group (b), AG group (c), and DMSO group (d), *n* = 10. The mice were sensitized by intraperitoneal injection of OVA (0.1 *μ*g/*μ*L) and intraperitoneally administrated AG (0.1 mg/ml) 1 h before each challenge with atomized OVA. Then, the mice were sacrificed and lung slices were analyzed by hematoxylin-eosin (HE) staining for observation of the neutrophil infiltration, bar = 250 *μ*m.

We investigated the inhibition of AG on the neutrophil infiltration in OVA-induced asthmatic mice model. As shown in Figure 4(b), OVA (0.1 *μ*g/*μ*L) treatment disorganized the epithelial cells, caused swelling and defluvium of part of the cells, and increased the neutrophil infiltration of lung tissue and inflammation of interstitial tissue in OVA-stimulated asthmatic mice, while the situation was gradually ameliorated by AG in Figure 4(c).

There was an AG inhibition of the airway remodeling in OVA-stimulated asthmatic mice.

**Figure 5:**
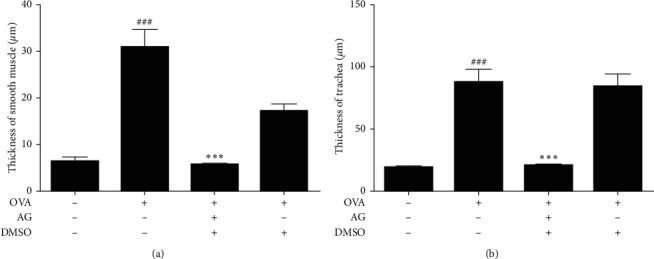
AG inhibition of the thickness of ASM and trachea in OVA-stimulated asthmatic mice. As shown in (a) and (b) OVA (0.1 *μ*g/*μ*L) treatment markedly increased the thickness of ASM and trachea, which were significantly abated by AG in the dosage of 0.1 mg/ml (*p* < 0.01). The values are expressed as the mean ± the standard deviation of triplicate tests. ^###^*p* < 0.01, relative to the control group; ^*∗∗∗*^*p* < 0.01, relative to the OVA group.

**Figure 6:**
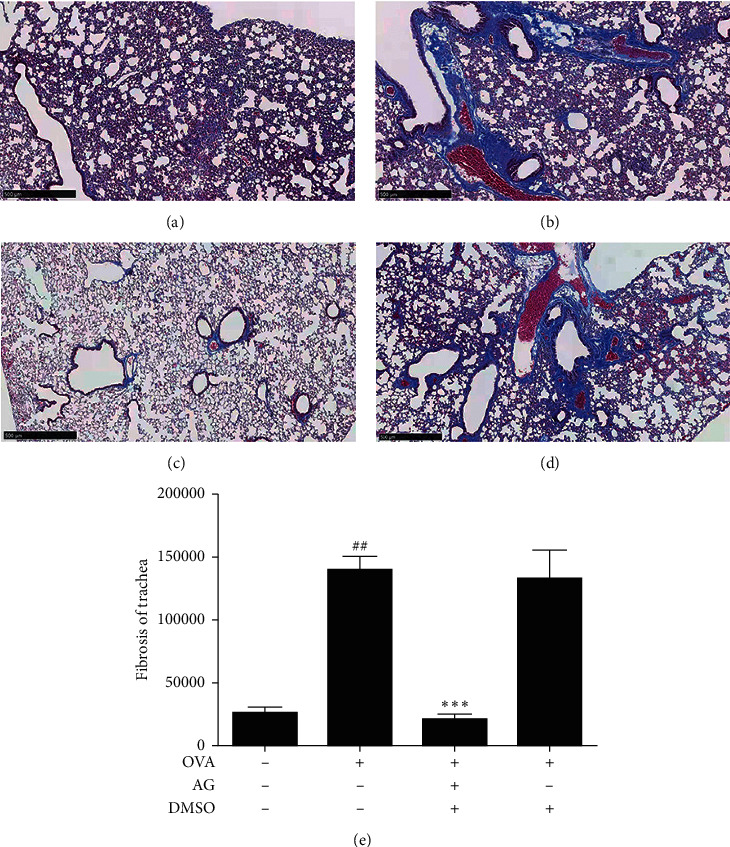
AG inhibition of the fibrosis of trachea in OVA-stimulated asthmatic mice. Animals were randomly divided into 4 groups: control group (a), OVA group (b), OVA + AG group (c), and OVA + DMSO group (d), *n* = 10. Then the lung slices were analyzed by Masson staining for observation of the fibrosis of trachea and the airway remodeling, bar = 500 *μ*m. The statistical bar was shown in (e). The values are expressed as the mean ± the standard deviation of triplicate tests. ^##^*p* < 0.05, relative to the control group; ^*∗∗∗*^*p* < 0.01, relative to the OVA group.

**Figure 7:**
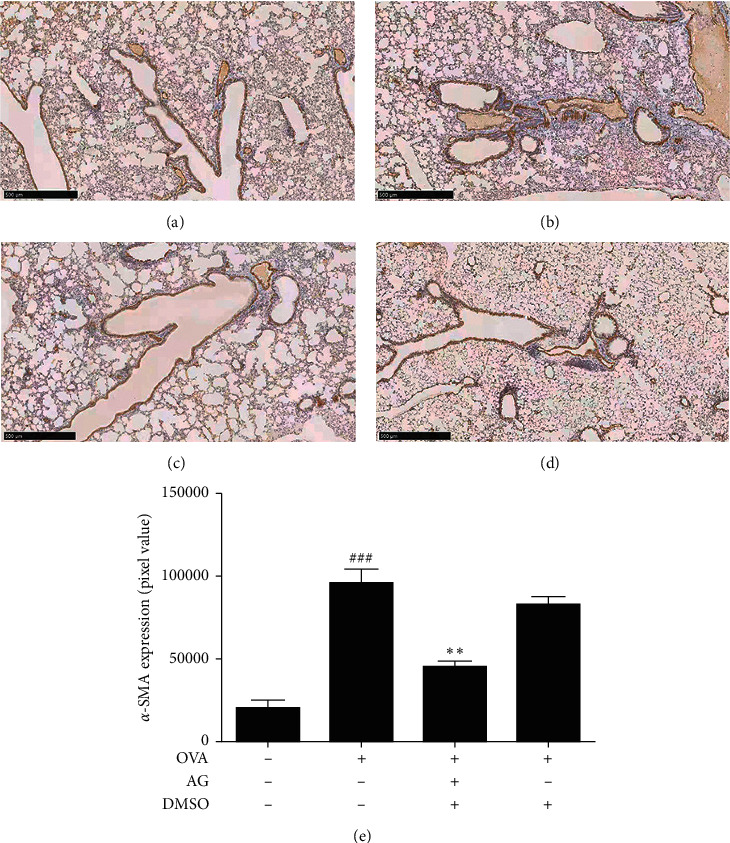
AG inhibition of the expression of *α*-SMA in OVA-stimulated asthmatic mice. Animals were randomly divided into 4 groups: control group (a), OVA group (b), OVA + AG group (c), and OVA + DMSO group (d), *n* = 10. The lung slices were analyzed by immunohistochemical staining for testing the expression of *α*-SMA, bar = 500 *μ*m. The statistical bar was shown in (e). The values are expressed as the mean ± the standard deviation of triplicate tests. ^###^*p* < 0.01, relative to the control group; ^*∗∗*^*p* < 0.05, relative to the OVA group.

Next, we explored the suppression of AG on airway remodeling in OVA-induced asthmatic mice model. As shown in Figures 5(a) and 5(b), OVA (0.1 *μ*g/*μ*L) treatment increased the thickness of ASM and trachea wall in OVA-stimulated asthmatic mice. However, the pathological change was significantly inhibited by AG (*p* < 0.01). As shown in Figure 6(b), OVA (0.1 *μ*g/*μ*L) treatment increased the fibrosis of trachea and collagen deposition in alveolar septum. Yet AG could significantly reduce the condition of fibrosis and collagen deposition in Figure 6(c). The statistical bar was shown in Figure 6(e) (*p* < 0.01). What is more, it shows in Figure 7(b) that OVA (0.1 *μ*g/*μ*L) stimulation increased the expression of *α*-SMA and irregularity of muscle tissues around the trachea. As shown in Figure 7(c), this condition was significantly abated by AG in ASM. The statistical bar was shown in Figure 7(e) (*p* < 0.05).

## 4. Discussion

Asthma is a heterogenous disease with the basic nature of airway inflammation. The chronic progression and intermittent exacerbation will eventually lead to pathological changes such as inflammatory cells infiltration, airway hyperresponsiveness, airflow obstruction, and airway remodeling. Allergic asthma is the most common asthma phenotype, and Th2 immunity is classically thought to be important in mediating bronchial inflammation during allergic asthma by cytokines such as IL-4, IL-5, and IL-13 produced from CD4 + Th2 cells and innate lymphoid cells [22]. However, only half of asthmatic patients with airway inflammation are observed to be mediated by Th2 cells [23]. Nonallergic asthma is another kind of important phenotype which refers particularly to the patients with non-Th2 inflammation, and one of the major mechanisms leading to a non-type 2 response is thought to result from the activation of the IL-17-mediated pathway [24]. Obviously it is of great significance to explore more comprehensive mechanisms of asthma.

As the subpopulation of T helper cells, Th17 cells produce IL-17 A/IL-17F and play a pivotal role in inflammation, autoimmunity, and host defense against extracellular pathogens [25, 26]. The fact that Th17 cells are more prone to react on the innate immune system, produce cytokines, and eventually activate the adaptive immune response demonstrates the essentiality that Th17-mediated immune responses not only regulate host defense but also promote chronic inflammation and autoimmunity [27]. It is concluded that comparing to mild/moderate asthma, IL-17 is increased in severe asthma, and the large amount of IL-17 production is an independent risk factor for severe asthma [28]. Importantly, research also showed the pathogenic role of IL-17 as the exacerbator of frequent asthmatic attacks by detecting the high expression of IL-17-related cytokines in bronchial/nasal mucosa of neutrophilic asthma [29]. It also indicates that IL-17 and neutrophils are proposed to play a role especially in those with severe asthma or asthma resistant to glucocorticoids [30], which would be explained by the effect of IL-17 in the microenvironment of the lung, including direct activation and possible recruitment of neutrophils to the airways [31]. Meanwhile, IL-6 is a typical proinflammatory cytokine which is produced following immune activation and is related to the pathogenesis of many inflammatory diseases. Elevated IL-6 not only promotes allergen-induced airway inflammation through progranulocytic cytokine and chemokine production but also associates with mixed eosinophilic/neutrophilic asthmatic airway inflammation and degenerated lung function [32]. As a specific marker of non‐T2 asthma, high plasma IL‐6 might be a potential therapeutic target in these asthmatics compared with other endotypes [33]. In our study, we found that AG inhibited the expression of IL-6, IL-17A, and IL-17F in both serum and BALF of OVA-stimulated mice. These results clearly confirmed the anti-inflammatory effects of AG.

ROR*γ*t and ROR*α* are transcription factors expressed in Th17 cells which have been considered to play a role in Th17 differentiation [34], while the function of ROR*γ*t is more evident. ROR*γ*t is especially indispensable in the differentiation of Th17 cells and transcription activation of IL-17 A/IL-17F [35]. The usage of ROR*γ*t inhibitors suppressed Th17 differentiation, IL-17 production, and afterwards Th17 cells-mediated diseases [36]. ROR*γ*t-deficient mice developed low-grade lung inflammation by less infiltration of inflammatory cells and compromised induction of inflammatory cytokines [37]. Our research also indicated the inhibitory effect of AG in the expression of both mRNA and protein levels of ROR*γ*t. These results strongly verified that AG could inhibit the Th17 cells-related transcription factor.

The mainly pathologic changes of asthma are airway remodeling, and it is closely related to the prognosis of asthmatic patients. The key features of airway remodeling are epithelial shedding, subepithelial fibrosis, increased smooth muscle cell mass, and hyperplasia of goblet cell and mucous gland [38]. It is considered that the high level of IL-17A not only alter ASM function and structure which continuously leads to airway remodeling, but also modulate interactions between subepithelial fibrosis and ASM to enhance airway remodeling in association with profibrogenic factors [39]. It is confirmed that in asthmatic fibrocytes, IL-17A induced a marked increase in the release of cytokines functioning in recruitment of neutrophil such as CXC chemokine ligand (CXCL) 8 and CXCL1, and the gene and protein expression of *α*-smooth muscle actin (SMA), a well-known inducer of contractile protein, was almost completely inhibited by the treatment of neutralizing antibody against CXCL8 in IL-17A-cultured fibrocytes [40]. Research has also witnessed the effect of anti-IL-17 treatment in asthmatic mice models in inhibiting pulmonary vascular remodeling, decreasing neutrophils in BALF, and relieving peribronchovascular*** *** edema [41]. Given the certified function of IL-17 in regulating airway structure in previous studies, we accordingly assume that one of the main pathomechanism of asthma was Th17 cells recruiting the neutrophils and regulating MF and ASM cells to aggravate the inflammation and airway remodeling. The results demonstrated in our study clearly proved the inhibitory properties of AG in pathological progress of OVA-induced asthmatic mice model.

In our research for the mechanism by which AG mediates the airway inflammatory response, we focused on the OVA-stimulated JAK1/STAT3 signaling pathway. JAK1 is a member of the Janus kinase family of proteins functioning with its downstream target protein STAT3 in initiating and mediating the inflammatory response. JAK1/STAT3 was suggested as the target pathway involved in hypertrophy of human bronchial smooth muscle and contributed to the increase in ASM mass and airway narrowing [42]. JAK1 inhibitor is sufficient to suppress asthma-related inflammation and lung pathology in OVA-induced animal models [43]. Moreover, STAT3 has been considered the potential target for asthma therapy. In in vivo studies, STAT3 could be activated to induce the differentiation of CD4 + T cells into Th17 cells which subsequently activated transcription factors ROR*γ*t and ROR*α* to produce IL-17A, IL-17F, and IL-22 [44, 45]. The intervention of STAT3 inhibitor could alleviate inflammation and remodeling of airway and the infiltration of Th17 cells [46]. STAT3 is also responsible for airway remodeling by facilitating epithelial-to-mesenchymal transition [47], involved in both the ASM tissues of patients with asthma and ASM cells under the exposure of asthma-related cytokines and observed to mediate the proliferation, migration, and tube‐forming ability of human lung microvascular endothelial cells [48]. Thus, we tested whether the anti-inflammatory effect of AG was relevant to the inhibition of JAK1/STAT3 activation in OVA-induced asthmatic mice. Our study showed that AG hampered JAK1 and STAT3 phosphorylation, suggesting that the JAK1/STAT3 pathway was suppressed by AG in activated asthmatic mice. These results illustrate the ability of AG to inhibit the inflammatory response via the JAK1/STAT3 pathway.

## 5. Conclusions

**Figure 8:**
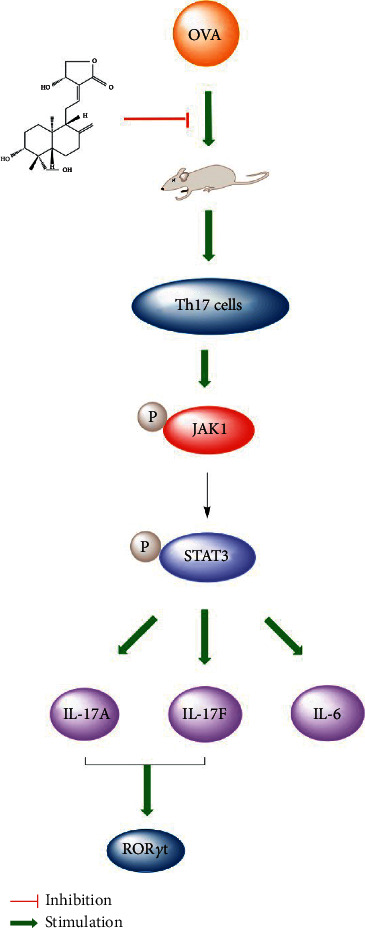
Proposed antiairway inflammatory mechanism of AG on OVA-stimulated asthmatic mice. OVA activates the expression of Th17-related cytokines and transcription factor via JAK1/STAT3 signaling pathway which could be suppressed by AG, demonstrating antiairway inflammatory effects in OVA-stimulated asthmatic mice.

In conclusion, our results demonstrate that AG can relieve neutrophil infiltration of lung tissue and airway remodeling. Meanwhile we also found that AG could suppress Th17-related cytokines by suppressing the activation of the JAK1/STAT3 pathway in OVA-stimulated asthmatic mice. The specific mechanism is outlined in Figure 8. Together, these findings provide expansive insight into future research. The suppressive effect of AG on JAK1/STAT3 signaling pathway by regulating Th17 cells may have the potential for treating asthma or other inflammation-mediated diseases.

## Figures and Tables

**Table 1:** AG inhibits the mRNA expression of RORγt. The PCR primer of mRNA is shown.

Primers	Sequences (5′to3′)
Mouse ROR*α* forward	GTGGAGACAAATCGTCAGGAAT
Mouse ROR*α* reverse	TGGTCCGATCAATCAAACAGTTC
Mouse ROR*γ*t forward	GAAGGCAAATACGGTGGTGT
Mouse ROR*γ*t reverse	AGAGGGCAATCTCATCCTCA
Mouse *ß*-actin forward	CATCCGTAAAGACCTCTATGCCAAC
Mouse *ß*-actin reverse	ATGGAGCCACCGATCCACA

## Data Availability

All relevant data are presented within the manuscript.
